# P-847. Antimicrobial Stewardship in a Hospital-at-Home Program: Impact on Antibiotic Duration and Clinical Outcomes in Patients with Community-Acquired Pneumonia

**DOI:** 10.1093/ofid/ofaf695.1055

**Published:** 2026-01-11

**Authors:** Charles Jensen, Sky R Blue, Cathy Hitt Piechowski, Cathy Oliphant, Lizveth Lopez

**Affiliations:** St. Luke's Health System, Boise, ID; St. Luke's Health System, Boise, ID; St. Luke's Health System, Boise, ID; St. Luke's Health System, Boise, ID; St. Luke's Health System, Boise, ID

## Abstract

**Background:**

Hospital-at-Home (HaH) programs face unique antimicrobial management challenges. We embedded our Antimicrobial Stewardship Program at the launch of a new HaH initiative to review all antimicrobials—an intensity not standard in traditional inpatient (TI) settings at our health system. We compared CAP outcomes between these care models.

**Figure 1.**
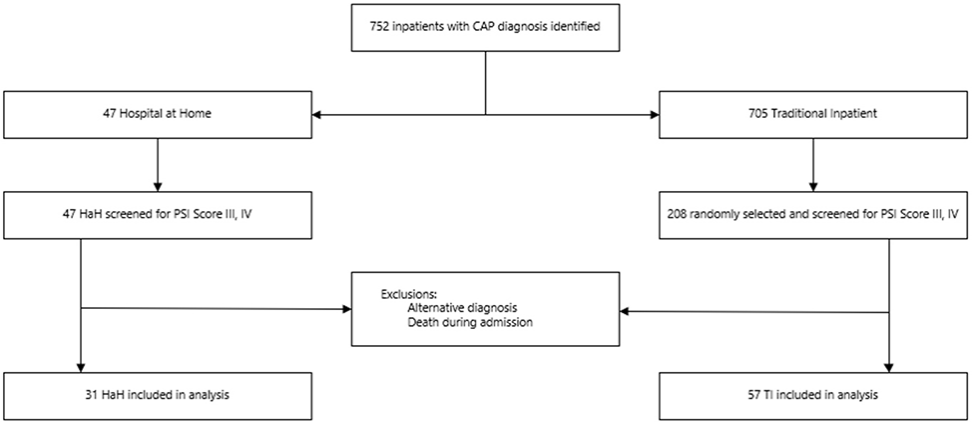
Patient Selection Flowchart

**Table 1.**
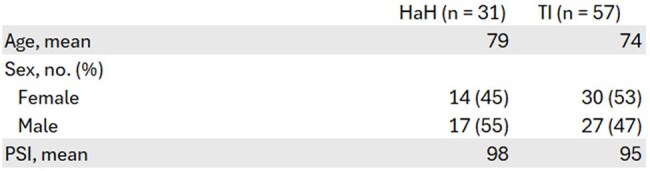
Baseline Characteristics Abbreviations: HaH, Hospital at Home; TI, Traditional Inpatient; PSI; Pneumonia Severity Index.

**Methods:**

We retrospectively compared adult CAP patients (PSI scores 71-130) treated in TI versus HaH from November 2024-April 2025. Patients with alternate diagnoses or in-hospital death were excluded. Patients were randomly selected at a 2:1 ratio. TI patients received standard stewardship triggered by duration thresholds, formulary restrictions, or drug-bug mismatches. HaH patients received intensive stewardship with daily antimicrobial review and multidisciplinary input.

**Table 2.**
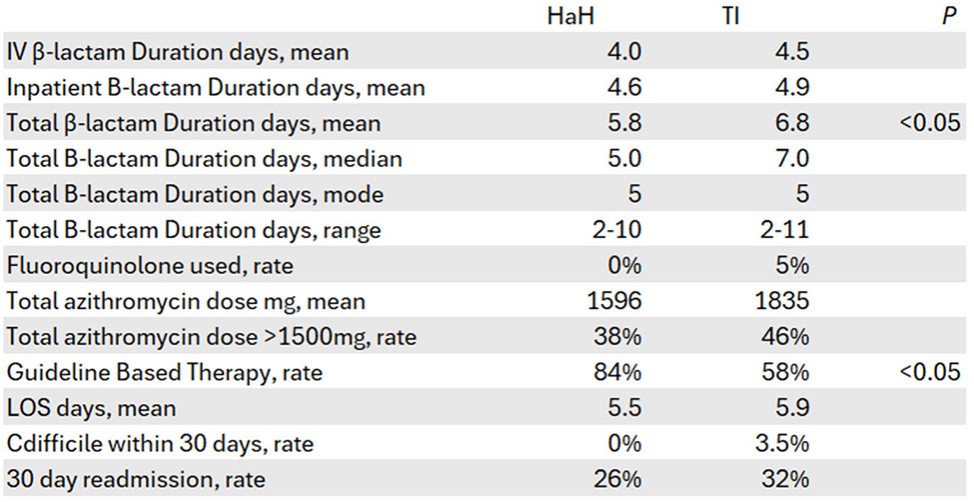
Outcomes Abbreviations: HaH, Hospital at Home; TI, Traditional Inpatient; CAP, Community Acquired Pneumonia; IV, Intravenous; LOS, Length of Stay.

**Figure 2.**
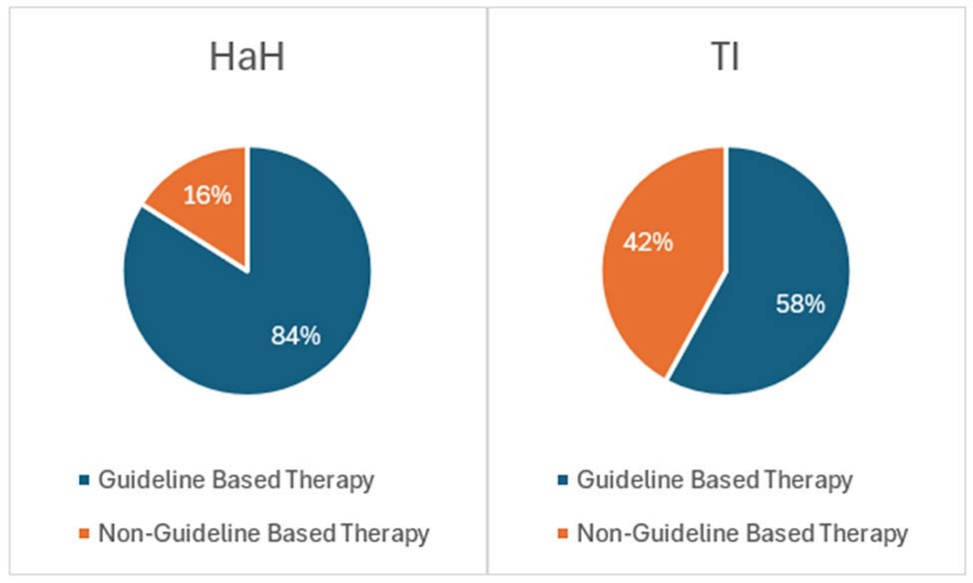
CAP Guideline Adherence Abbreviations: HaH, Hospital at Home; TI, Traditional Inpatient.

**Results:**

From 752 screened inpatients, 31 HaH and 57 TI patients met criteria. HaH patients showed significantly higher guideline adherence (84% vs 58%; p< 0.05) and shorter β-lactam therapy duration (5.8 vs 6.8 days; p< 0.05). HaH also trended toward shorter IV duration (4.0 vs 4.5 days), lower fluoroquinolone use (0% vs 6%), and lower excess azithromycin exposure (38% vs 58%), though these differences weren't statistically significant. Clinical outcomes (length of stay, Clostridioides difficile infection, and 30-day readmission) remained equivalent between groups.

**Conclusion:**

Intensive antimicrobial stewardship in HaH was well received by providers and significantly improved prescribing practices. These findings support intensive stewardship in HaH to reduce antibiotic exposure while maintaining outcomes. Applying this approach to the broader inpatient population during the study period would potentially eliminate 233 IV antibiotic days and 466 total antibiotic days.

**Disclosures:**

All Authors: No reported disclosures

